# Nondestructive circadian profiling of starch content in fresh intact Arabidopsis leaf with two-photon fluorescence and second-harmonic generation imaging

**DOI:** 10.1038/s41598-022-20618-5

**Published:** 2022-10-03

**Authors:** Juo-Nang Liao, Wei-Liang Chen, Chao-Yuan Lo, Man-Hong Lai, Huang-Lung Tsai, Yu-Ming Chang

**Affiliations:** 1 Center for Condensed Matter Sciences, National Taiwan University, Taipei, 10617 Taiwan; 2 Institute of Molecular and Cellular Biology, National Taiwan University, Taipei, 10617 Taiwan

**Keywords:** Plant sciences, Optics and photonics

## Abstract

Plant chloroplasts conduct photosynthesis to convert solar energy into sugars for the carbon source essential for cell living and growth during the day. One fraction of photosynthetic products is stored in chloroplasts by forming starch granules to continue the provision of carbon energy during the night. Currently, profiling the starch temporal pattern requires either: (i) sacrificing the leaves, or (ii) generating transgenic plants at the risk of changing the metabolisms by incorporating a genetically modified granule-bound starch synthase (GBSS). In this paper, we demonstrated a nondestructive method using two-photon fluorescence (TPF) and second-harmonic generation (SHG) imaging to quantify starch granules within chloroplasts of fresh intact leaves across a day-night cycle. We did so using two Arabidopsis lines having normal and excess starch contents: wild-type (Columbia-0) and *starch excess 1* (*sex1*). The starch granules were visualized by SHG imaging, while the chloroplasts in mesophyll cells were visualized by TPF imaging. Our results provided micron scale spatial resolution of starch distribution within leaves and showed starch circadian patterns consistent with those profiled by enzymatic assays in previous studies. We demonstrated that TPF-SHG imaging is a potential tool for revealing the real-time heterogeneity of starch circadian rhythm in leaf cells, without the need for destructive sample preparation.

## Introduction

Starch is the main storage of carbohydrates in higher plants^1^. There are two primary places where starch is produced in plants. One is in the leaves where photosynthesis takes place. The other is in the storage organs such as the stems and roots. The starch in the storage organs accumulates continuously and is not degraded until it is needed for growth. Starch in the leaves, however, has a diurnal variation. During the day, photosynthesis produces starch that accumulates in the chloroplast then forms into starch granules^2,3^. When photosynthesis ceases at night, the energy needed for cell survival and growth is provided by the degradation of starch stored in the leaves during the day^4–6^. The ability to monitor immediate starch variations is important for tracking diel mechanisms involved in starch metabolism.

Methods commonly used for starch quantification include enzymatic methods^7,8^, starch-iodine staining, and genetic modification. Enzymatic methods extract and degrade starch with enzymes and can be used to quantify the total amount of the glucose residue stored in starch^9^. The iodine solution staining method uses iodine to dye the starch without extracting it from the leaf. But to observe the stained starch clearly, the sugars and chlorophyll need to be dissolved and removed with alcohol. This method allows determination of the starch position and distribution in the tissue, but due to the requirement for dissolving the chlorophyll and staining with iodine solution, the method destroys the leaves during the preparation process. Another method for starch determination is by cloning a construct that contains a target gene of interest, then obtaining the transgenic plants that carry the construct randomly integrated into the genome. Using an enzyme that participates in starch metabolism and fusing it to a fluorescent protein results in a transgenic plant that allows the observation of starch through fluorescent imaging^10,11^. Although utilizing fluorescent protein imaging for quantitative starch analysis does not destroy the transgenic leaf, creating a transgenic plant is complicated and time-consuming, and needs to be done for each ecotype of interest. Another non-destructive method that had been used to quantify starch in plants is microCT imaging, which was applied specifically to the ray and axial parenchyma (RAP) in woody stems^12^. Because this last method does not provide unique contrast from starch, it relies on using the shapes and sizes of the RAP cells from image analysis, and cannot easily be applied generally for starch determination.

Since starch granules are semicrystalline^13–15^, a possible method for its identification is by second harmonic generation (SHG)^16,17^. SHG is a nonlinear optical process that is allowed only in non-centrosymmetric structures and has been used as a contrast mechanism in multiphoton microscopy for imaging a variety of intact biological tissues such as bones, tendons, and cartilage^18–20^. It has also been shown to provide contrast for imaging starch and cellulose in plants ^21–23^. For SHG starch studies, research groups have mostly analyzed starch structure by using SHG detection after the starch had been extracted^24–26^. The SHG images acquired usually relied solely on the use of spectral filters to separate the SHG signals from the fluorescence. Without spectral confirmation, wide bandwidth fluorescence signals close to the SHG spectral band can leak through the SHG filter and be detected as SHG intensity, resulting in a misrepresented SHG image. In this study, we used SHG imaging to profile the starch content of intact Arabidopsis leaves throughout a 24-h light–dark cycle. SHG imaging in leaf cells was subcellularly colocalized with TPF signals emitted by chloroplasts, suggesting that the SHG imaging was generated by starch granules. We developed a method based on SHG and TPF intensity images to quantify the starch content inside the chloroplast. Furthermore, spectral mapping of the sample further confirmed the SHG images faithfully represented the SHG intensity variations. We demonstrated the effectiveness of our method to monitor nondestructively starch variations using two kinds of Arabidopsis: One wild-type Columbia-0 (Col-0) with normal starch production, and one *sex1* mutant with a defective SEX1 that prevents starch degradation and utilization, resulting in an excess of starch accumulation in the chloroplast. We observed that the SHG imaging signal increased during the day and decreased during the subsequent night in Col-0 leaves but remained consistently high in *sex1* leaves. The different variations of SHG under diel cycles between Col-0 and *sex1* demonstrated that the fluctuation of starch granules in living cells can be effectively detected with SHG imaging.

## Results and discussion

### Multiphoton laser scanning images of chloroplasts inside a fresh leaf

**Figure 1:**
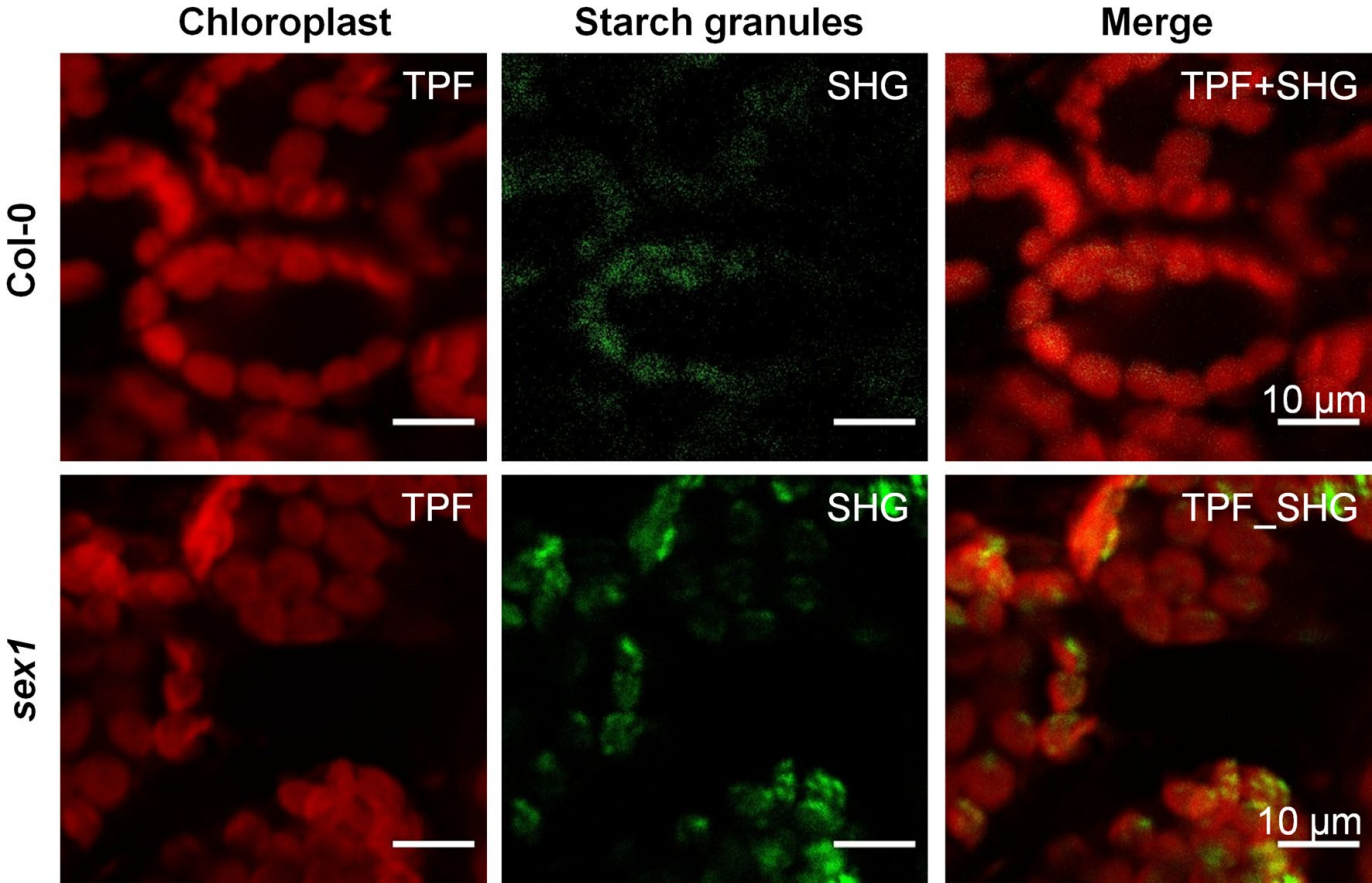
Two photon fluorescence (TPF) and second harmonic generation (SHG) images of fresh intact wild-type Columbia-0 (Col-0) leaf and fresh intact *starch excess 1* (*sex1*) mutant leaf. The last column shows the combined TPF and SHG images. The TPF images were acquired with a 670/30 nm filter, while SHG images were acquired with a 532/3 nm filter. The pixel dwell time was 0.016 ms and 0.128 ms for the TPF and SHG image acquisitions, respectively.

Figure 1 shows the TPF and SHG images of fresh intact Col-0 and *sex1* leaves. From the TPF images, contrast from the chlorophyll autofluorescence allows visualization of chloroplasts in mesophyll cells. It can be seen that the autofluorescence does not completely fill the chloroplasts and there are many dark sites. The middle column shows the SHG images from the same scanning area, where the SHG signal is expected to come from the starch granules. In the third column, merged TPF and SHG images show that the SHG appears inside the chloroplasts, in the areas where the autofluorescence signal is weak. This is expected since starch granules occupy spaces in the stroma, while the chlorophyll is restricted at the thylakoid membranes, resulting in the spatial separation of the TPF and SHG signals. Furthermore, we found that SHG signals in the *sex1* leaf are stronger than those in the Col-0 leaf, consistent with the excess starch known to be present in the *sex1* mutant.

### SHG spectrum in fresh leaves

**Figure 2:**
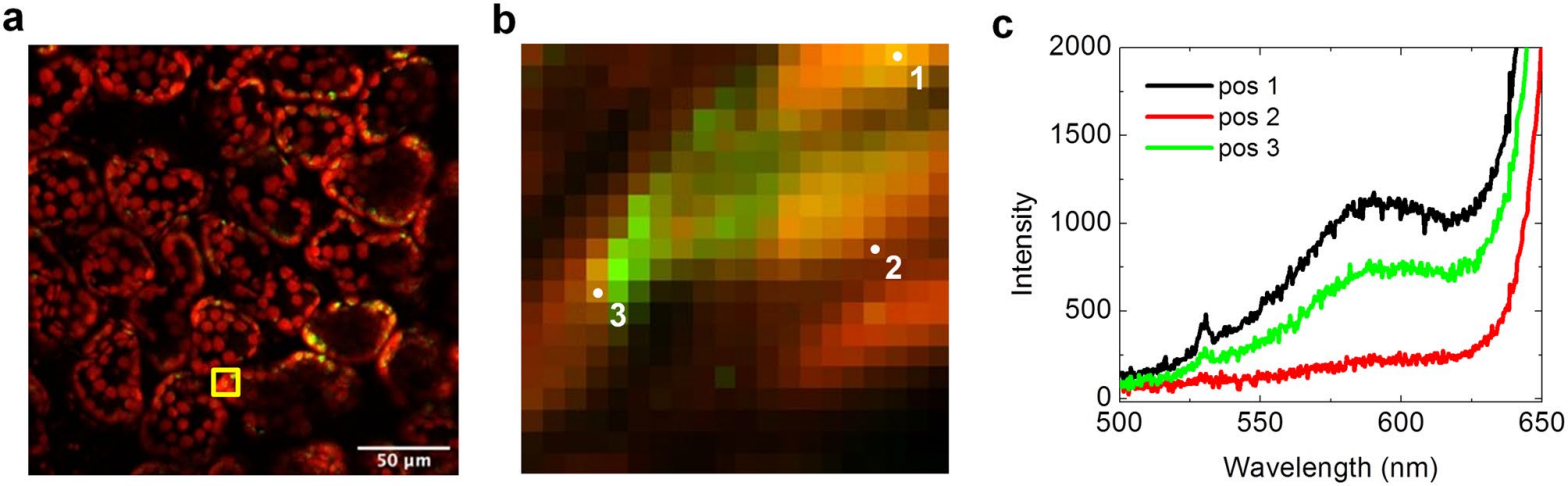
Spectral mapping for a fresh Col-0 leaf. (**a**) TPF (red) and SHG (green) imaging of a fresh intact wild-type Col-0 leaf. (**b**) Spectral mapping of the yellow box area in (**a**), with the total spectral intensity of the 655–685 nm band shown in red and the 530.5–533.5 nm band shown in green. (**c**) Selected spectra from the spectral mapping with 5 s integration, with the SHG peak feature visible.

**Figure 3:**
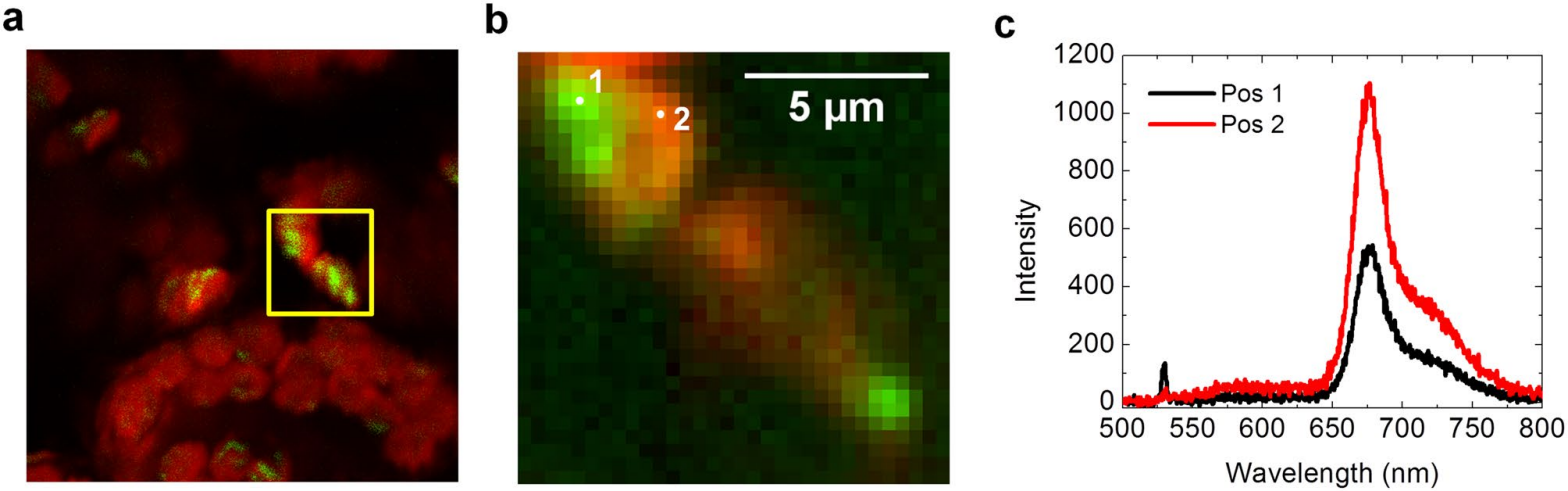
Spectral mapping for a fresh intact *sex1* mutant leaf. a TPF (red) and SHG (green) imaging of a fresh intact *sex1* mutant leaf. b Spectrum mapping of the yellow boxed area in (**a**), with the total spectral intensity of the 655-685 nm band shown in red and the 530.5–533.5 nm band shown in green. (**c**) Spectrum of selected points shown in (**b**) with 0.3 s integration. The Spectrum from point 1 show a SHG peak feature with a lower chlorophyll fluorescence.

To confirm that the PMT images acquired with the SHG and TPF bandpass filters correspond to the spatial distribution of starch SHG and chlorophyll TPF, we acquired a spectral mapping in a selected region of the PMT mapping. We first obtained the combined TPF and SHG PMT image of a fresh intact Col-0 leaf in the NDD configuration (Fig. 2a). Then we switched to the descanned detection and acquired a spectral mapping of the yellow square area shown in Fig. 2a, with a spectral integration time of 5 s. For each spectrum in the spectral mapping, we summed the spectral intensities over the wavelength bands corresponding to the SHG and TPF filters used for the PMT mapping. Figure 2b shows the resultant image with the summed intensities over the SHG and TPF filter wavelength bands represented in green and red respectively. The image corresponds to the yellow square area in Fig. 2a, acquired in the descanned confocal configuration. Figure 2c shows a few selected spectra corresponding to the marked positions in Fig. 2b. From these spectra, we see that in a typical image pixel, both the SHG spectral peak feature at 532 nm and a large spectral rise due to chlorophyll autofluorescence are present. Besides chlorophyll fluorescence and the starch SHG, we see a small additional unidentified fluorescence feature around 590 nm. Although this fluorescence feature is small compared to the chlorophyll fluorescence, its fluorescence tail can make a small contribution to the 532 nm SHG band in the PMT image. Figure 3 shows similar imaging and spectral mapping for a fresh *sex1* leaf, but with a shorter spectral integration time of 0.3 s. Due to the greater amount of starch present in the *sex1* mutant, the shorter integration time can produce spectra with SHG intensities comparable to that of the Col-0 wild type. These spectral observations provide confirmation that the SHG filtered PMT images correspond to the SHG intensity, allowing us to use the SHG intensity to quantify the amount of starch present in the leaf. The greater SHG intensities observed in *sex1* are consistent with the fact that *sex1* mutants lead to greater starch accumulation in the chloroplast than the Col-0 wild types.

### Monitoring of diurnal variations of starch granule in Arabidopsis

**Figure 4:**
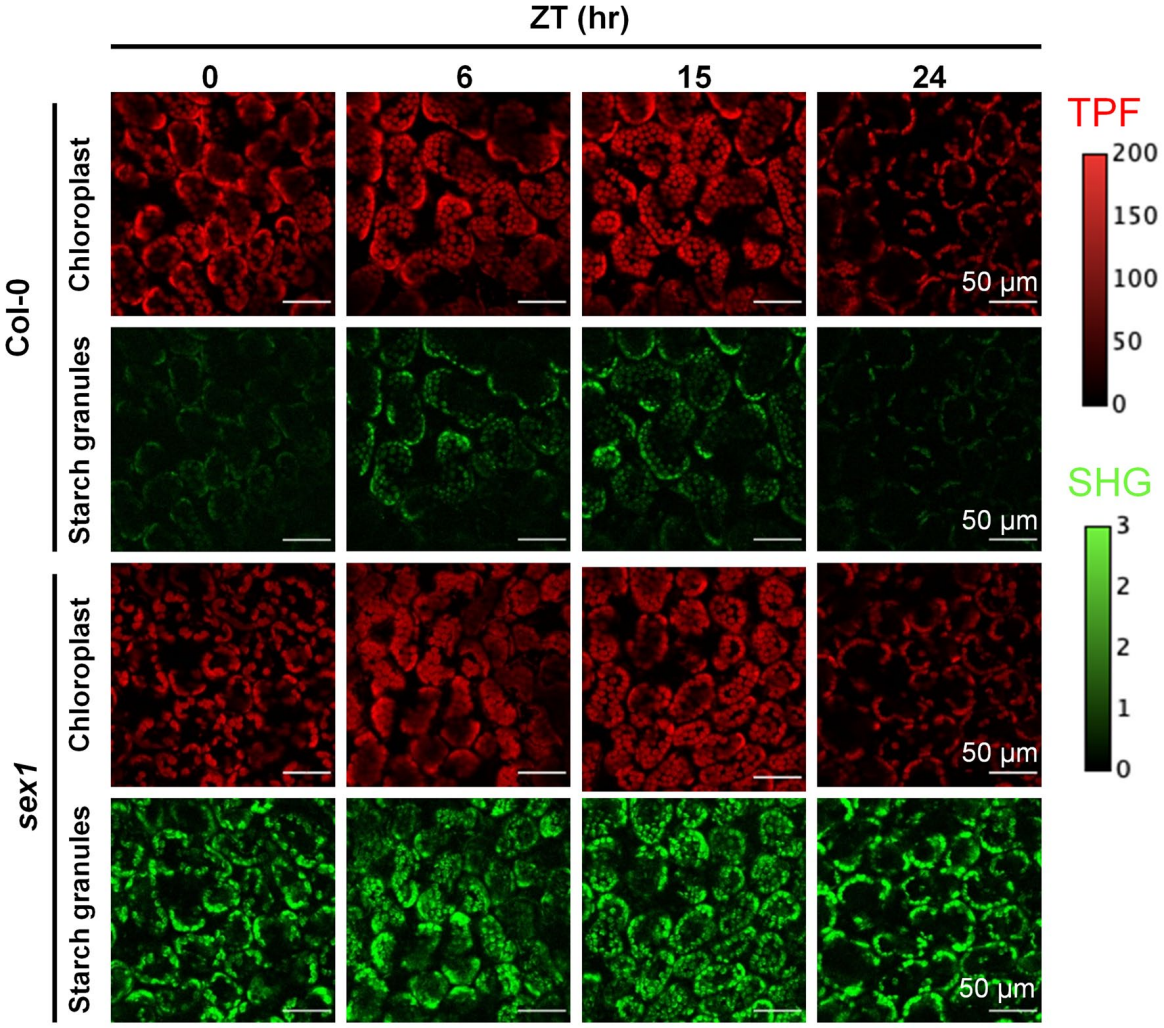
Diurnal variations of starch granule in Arabidopsis with TPF-SHG imaging. TPF (red) and SHG (green) imaging of fresh intact Col-0 and *sex1* leafs at different Zeitgeber Time (ZT) hours. For Col-0, variations in the SHG intensity can be seen for the different ZT hours.

**Figure 5:**
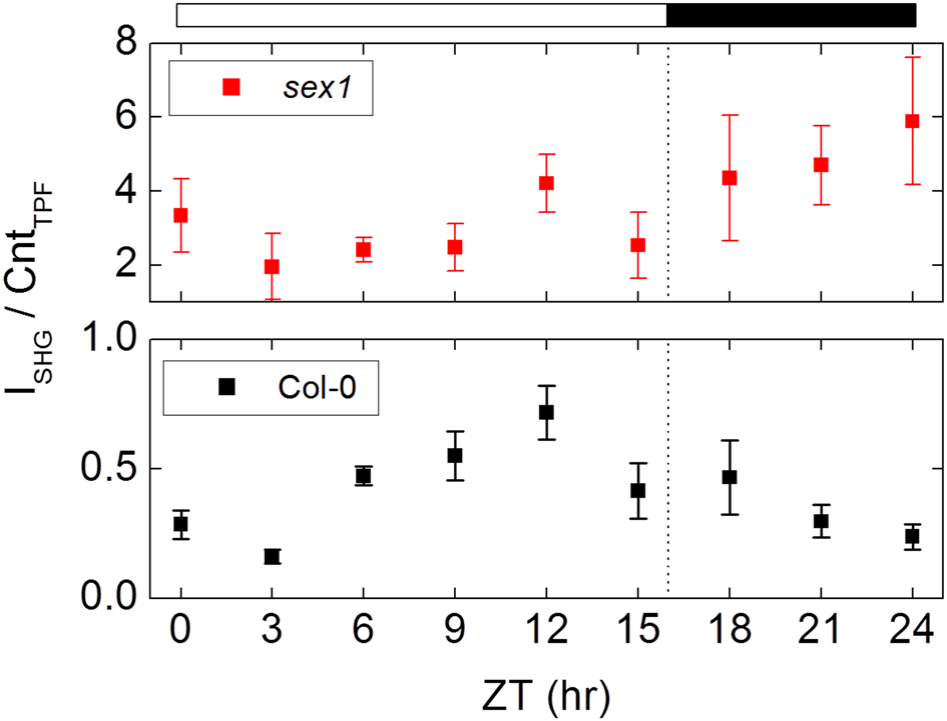
The ratio of SHG intensity to TPF pixel counts above threshold in the images were analyzed and plotted across a 16-h light/8-h dark diurnal cycle. The horizontal axis shows ZT hours, starting at 0 h after the light comes on (ZT0) and increments in 3 h intervals until ZT24. The bar on top indicates the hours when the growth chamber light was on and off, and the dashed line indicates ZT16 hour when the light in the growth chamber was turned off. Error bars correspond to one standard deviation based on 12 imaging areas of two leaves. (I_SHG_: summed intensity for pixels above SHG threshold of 2; Cnt_TPF_: total pixels above TPF threshold of 50).

As a test of our system's applicability to monitoring the starch content in fresh intact leaves, we designed an experiment to monitor the diurnal variation of starch granules. It is known that starch granules in leaves are synthesized through photosynthesis during the day and degrade at night to provide energy to the plant during the dark hours. Figure 4 shows the sequence of TPF and SHG images in Col-0 or *sex1* leaves across a 16-h light/8-h dark cycle. The intensity values correspond to the detected photon count during the pixel integration intervals. We acquired images at time intervals of 3 h during the light–dark cycle and calculated the ratio of SHG intensity to TPF pixel count (Fig. 5). Since the starch SHG signal only comes from within the chloroplast, we used the number of TPF pixels to quantify chloroplast areas in an image. The SHG intensity to TPF pixel ratio therefore represents a relative quantity of starch inside chloroplast for different ZT hours. In Fig. 5, the horizontal axis represents ZT hours, starting at 0 h after the lights turn on (ZT0) and proceeding at three-hour intervals until ZT24. The ratio represents the variations of the leaf starch content throughout a day. The ratio in the Col-0 leaves indicates that the starch content increases due to photosynthesis during the light hours and decreases during the dark hours as the starch is degraded to provide energy. Although the *sex1* mutant shows a greater starch content throughout the light–dark cycle, its defective degradation of starch leads to no obvious downward trend for the starch content during the dark hours. A small non-zero background observed in the ratio for Col-0 shows some amount of starch remains at dawn but may also be attributed to a small TPF leakage into the SHG detection band (Fig. 2c). Our data for the two Arabidopsis lines show starch patterns similar to those profiled by enzymatic assays^27^, without lengthy sample preparation or destruction of the observed leaves. TPF-SHG imaging therefore provides a minimally invasive method for monitoring the starch content within a leaf with the potential for real-time in situ monitoring of a leaf over multiple circadian cycles.

## Conclusion

Current methods for profiling leaf starch content for diurnal studies of plants require either generating transgenic plants or sacrificing the observed leaves. By using TPF and SHG imaging to monitor fresh leaf starch content of two kinds of Arabidopsis, Col-0 and *sex1* mutant, over a 24-h light–dark cycle, we have successfully demonstrated the capability to quantify nondestructively starch content for diurnal studies of fresh leaves. The TPF imaging allowed visualization of chloroplast while the SHG imaging provided spatial distribution of starch. Spectral acquisition further confirmed the origin of TPF and SHG signals. Since this method was done on fresh intact leaves with minimal sample preparation, it can potentially be used for real-time in situ imaging. Our method allows the study of starch circadian rhythm in not only a single leaf but also different parts of a leaf down to a single cell. Thus, starch in different cell types, cells in different developmental zones, or any stimulus on changing the leaf starch diurnal variations can potentially be studied in real-time.

## Methods

### Plant materials (Col-0, *sex1*) and growth condition

The seeds of wild type Columbia-0 (Col-0) and the *sex1-1* mutant (germplasm stock: CS3093 is publicly available in Arabidopsis Biological Resource Center “https://abrc.osu.edu/”) were kindly provided by Jychian Chen from the Institute of Molecular Biology, Academia Sinica, Taipei^28^. All plant studies were performed in accordance with the relevant guidelines and regulations provided by the authors’ respective institutions and the Taiwan Centers for Disease Control. We first sowed the bleach-cleaned Col-0 or *sex1-1* seeds on half-strength Murashige and Skoog **(**1/2 MS) with vitamins (M519, PhytoTech LABs) in phytoagar plates, and placed the plates in a growth chamber (CH202, CHIN HSIN). The seeds were then incubated in a long-day (LD) photoperiods of 16-h light/8-h dark at 22 °C for 7–24 days. Just prior to imaging, we removed the plate from the chamber at the specific Zeitgeber Time (ZT) and detached the largest leaf from the seedling. The leaf was then directly placed on a glass slide, kept moist with drops of water, and covered with cover glass for imaging. For the starch diurnal variation experiment, we put the plates with sowed seeds in two separate growth chambers (CH202, CHIN HSIN), with the photoperiod initial time separated by 12 h. This allowed us to acquire data at ZT from 0 to 24 h in a half day period. We obtained images for ZT starting at 0 h when the light first came on and proceeded with 3 h intervals until ZT24.

### Multiphoton hyperspectral microscope system

The TPF and SHG imaging were performed on a custom-built laser scanning system (Photonic Workshop, LSCM 4.0). This system allowed for SHG and TPF imaging in the non-descanned detection (NDD) configuration and hyperspectral imaging in the descanned confocal detection (DD) configuration. The laser source was a 300-fs pulsed laser with a repetition rate of 40 MHz and a central wavelength of 1064 nm (Fianium, FemtoPower 1060-532-s). A 20X objective lens (Nikon, NA 0.75) focused the laser light with an on-sample power of 23–27 mW corresponding to a power intensity of 10 mJ/s‧μm^2^. A 670/30 nm filter was used for imaging chlorophyll TPF and a 532/3 nm filter was used for SHG imaging. For hyperspectral imaging, the signal was collected in the DD configuration by a 200 μm core fiber, then sent to a fiber-coupled spectrometer (Photonic Workshop, MR-SPEC), containing a 300 grooves/mm grating and a TE-cooled CCD (Andor, iDus).

### Data analysis

Since the starch granules are produced in the chloroplast of the mesophyll cells, we quantified the starch content by calculating the ratio of SHG intensity, I_SHG_, to the TPF pixels count, Cnt_TPF_, above a threshold for each image data. The SHG intensity was summed over only those pixels with a SHG count above the background level of 2, for the integration time of 0.128 ms. For the TPF pixel count, we used a threshold level of 50. This value was determined empirically to best delineate the chloroplast morphology that is in the focal plane of the image (See Supplementary Information for more details).

## Supplementary Information

Supplementary Information.

## Data Availability

The datasets generated and/or analyzed during the current study are available from the corresponding author at reasonable request.
