# Supplementary material for: Nondestructive circadian profiling of starch content in fresh intact Arabidopsis leaf with two-photon fluorescence and second-harmonic generation imaging
Source: Sci Rep. 2022 Oct 3;12:16525. doi: 10.1038/s41598-022-20618-5 (PMC9530172; doi:10.1038/s41598-022-20618-5)
Supplement: Supplementary file 1 — Supplementary Information. [file 41598_2022_20618_MOESM1_ESM.pdf]

Supplementary Information to Nondestructive circadian profiling of starch content in fresh intact Arabidopsis leaf with two-photon fluorescence and second-harmonic generation imaging.

Juo-Nang Liao[1,2], Wei-Liang Chen[1], Chao-Yuan Lo[1], Man-Hong Lai[1], Huang-Lung Tsai[2], Yu-Ming Chang[1]

[1]Center for Condensed Matter Sciences, National Taiwan University, Taipei 10617, Taiwan

[2]Institute of Molecular and Cellular Biology, National Taiwan University, Taipei 10617, Taiwan

Empirical determination of threshold value for pixels containing chloroplast

Fig. S1 compares the TPF images threshold at different values. The first column shows TPF images of Col-0 leaves at ZT0, ZT6, ZT15, and ZT21 with intensity scale set to 0 - 200. The columns to the right are the same image displayed as a binary image with the threshold value shown on top. A threshold value set too high leads to unaccounted pixels containing chloroplast, while a threshold value set too low would over count the area covered by chloroplast. Based on the images, we chose a threshold value of 50 to determine the number of pixels containing chloroplast. The sum of the pixels above the threshold value is the Cnt_{TPF} value used to calculate I_{SHG}/Cnt_{TPF} in Fig. 5 of the main text.

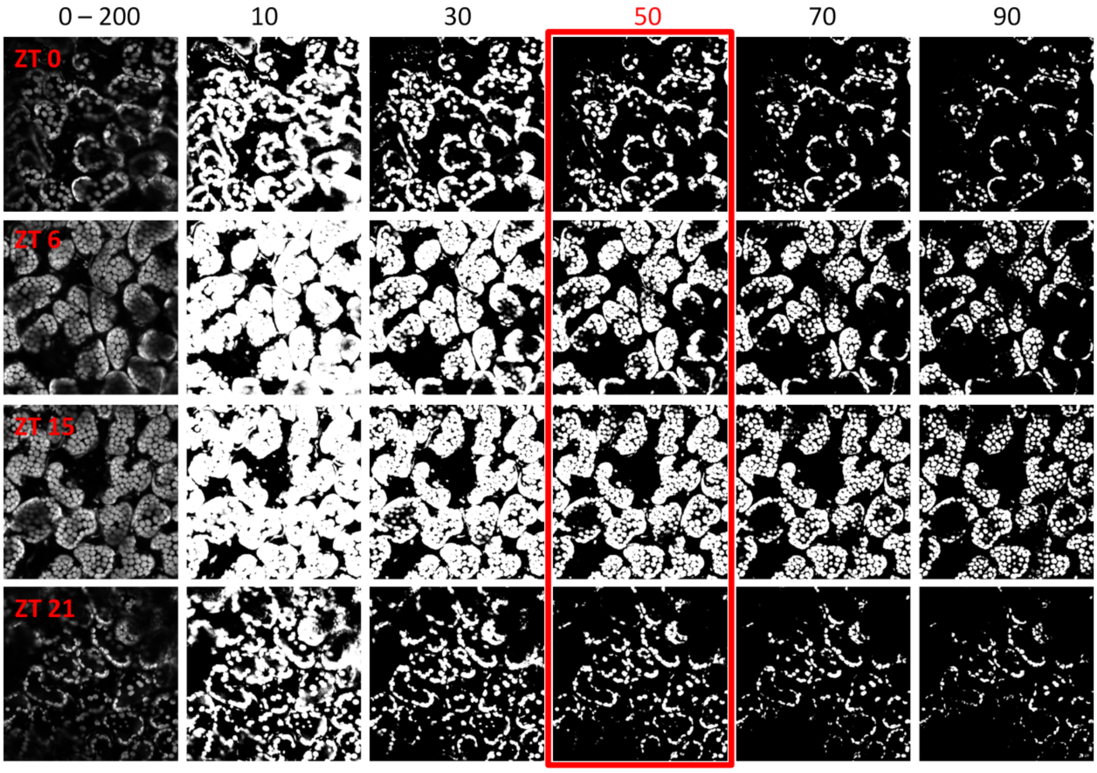

Figure S1 Empirical determination of TPF count threshold for statistical analysis.
